# High IL‐10 levels during CRS are negatively associated with NK‐cell recovery after CAR‐T cell therapy

**DOI:** 10.1002/cti2.70116

**Published:** 2026-07-24

**Authors:** Xindi Wang, Wenjing Luo, Yingying Li, Jianghua Wu, Lu Tang, Chenggong Li, Qiaolin Liu, Zhihan Chen, Yu Hu, Heng Mei

**Affiliations:** ^1^ Institute of Hematology, Union Hospital, Tongji Medical College Huazhong University of Science and Technology Wuhan China; ^2^ Hubei Clinical Medical Center of Cell Therapy for Neoplastic Disease Wuhan China; ^3^ Key Laboratory of Biological Targeted Therapy (Huazhong University of Science and Technology) Ministry of Education Wuhan China

**Keywords:** apoptosis, chimeric antigen receptor T‐cell therapy, cytokine release syndrome, ferroptosis, interleukin‐10, NK‐cell recovery

## Abstract

**Objectives:**

Immune reconstitution following chimeric antigen receptor (CAR)‐T cell therapy remains a critical clinical challenge, and the determinants of natural killer (NK)‐cell recovery are not fully understood.

**Methods:**

We longitudinally monitored natural killer (NK)‐cell recovery in 64 patients during the first year after CD19‐directed CAR‐T cell therapy, analysing NK‐cell counts and surface receptor expression. Associations between cytokine release syndrome (CRS), cytokine profiles, and NK‐cell numerical reconstitution were evaluated, and mechanistic insights were explored using *in vitro* NK‐cell assays.

**Results:**

NK‐cell numerical and phenotypic recovery was impaired within the first month after CAR‐T cell infusion. Notably, NK‐cell numerical recovery was significantly delayed in patients who developed CRS. A reduced NK‐to‐T cell ratio at one‐month post‐infusion was associated with a markedly increased risk of viral infection (hazard ratio = 4.512). Elevated interleukin (IL)‐10 levels during CRS were inversely associated with NK‐cell recovery. Patients with high IL‐10 levels exhibited delayed NK‐cell reconstitution, which was independently validated in two external cohorts. Mechanistically, *in vitro* exposure of NK cells to IL‐10 promoted caspase‐3 activation, increased apoptosis, and enhanced reactive oxygen species accumulation and lipid peroxidation. Rescue experiments using ferrostatin‐1 and Z‐VAD‐FMK further supported the involvement of IL‐10 in apoptosis and ferroptosis.

**Conclusion:**

These findings highlight a previously underappreciated role of IL‐10 in shaping NK‐cell reconstitution post‐CAR‐T cell therapy, particularly in the setting of CRS. Patients with CRS accompanied by elevated IL‐10 levels may benefit from anti‐inflammatory interventions or therapeutic strategies aimed at promoting NK‐cell recovery.

## Graphical abstract

This study demonstrates that high IL‐10 levels during cytokine release syndrome significantly delay natural killer (NK)‐cell recovery after CD19 CAR‐T cell therapy, increasing viral infection risks. Mechanistically, IL‐10 impairs NK‐cell recovery by promoting apoptosis and ferroptosis, suggesting that targeting IL‐10 or employing anti‐inflammatory interventions could improve NK‐cell reconstitution and patient outcomes.

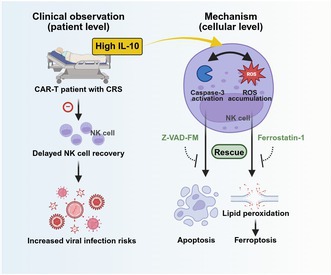

## Introduction

Chimeric antigen receptor (CAR)‐T cell therapy has revolutionised the treatment of haematological malignancies and immune‐related disorders.^1^, ^2^ Despite its remarkable therapeutic potential, this modality is often accompanied by adverse events such as cytokine release syndrome (CRS) and immune effector cell‐associated neurotoxicity syndrome (ICANS).^3^, ^4^ In addition to these complications, post‐treatment immune reconstitution represents a critical aspect that warrants careful consideration.^5^

Natural killer (NK) cells, as key components of the immune defence system, play essential roles in the elimination of tumorigenic and virus‐infected cells through both innate and adaptive immune mechanisms, contributing significantly to immunosurveillance.^6^ The phenotypic and functional diversity of NK cells can vary across different physiological and pathological contexts.^7^ In the setting of haematopoietic stem cell transplantation, NK‐cell reconstitution is influenced by factors such as interleukin (IL)‐15 and graft‐versus‐host disease (GVHD).^8^, ^9^ Favourable NK‐cell recovery has been correlated with reduced incidences of cytomegalovirus (CMV) infection/reactivation, disease relapse and chronic GVHD.^9^, ^10^, ^11^ Additionally, in patients with COVID‐19, expansion of unconventional CD56^dim^CD16^neg^ NK‐cell subset has been observed.^12^ However, the dynamics and clinical relevance of NK‐cell recovery in the context of CAR T‐cell therapy remain poorly understood. We hypothesise that NK‐cell profiles in patients undergoing CAR T‐cell therapy are heterogeneous, shaped by factors such as lymphodepleting (LD) chemotherapy, CAR T‐cell expansion, and therapy‐associated toxicities, and that these alterations may be prognostically significant.

The objective of this study was to characterise the dynamic changes in NK‐cell recovery following CD19 CAR T‐cell therapy in two clinical trials (NCT04008251 and NCT05765006). We observed that NK‐cell reconstitution was significantly delayed in patients who developed CRS compared to those who did not, potentially because of apoptosis and ferroptosis signalling mediated by elevated IL‐10 levels. Moreover, a reduced NK/T‐cell ratio appeared to be associated with an increased risk of viral infection or reactivation. These findings highlight the need for greater attention to NK‐cell recovery—particularly in patients with CRS—and support the development of novel strategies aimed at enhancing NK‐cell reconstitution following CAR‐T cell therapy to mitigate infection‐related complications.

## Results

### Patient characteristics

A total of 64 patients were included in this study, comprising individuals diagnosed with B‐cell acute lymphoblastic leukaemia (B‐ALL, 29.69%), non‐Hodgkin lymphoma (NHL, 51.56%, including diffuse large B‐cell lymphoma, follicular lymphoma, and mantle cell lymphoma) and systemic lupus erythematosus (SLE, 18.75%). The median age was 46 years (range, 18–80) and 53.13% of patients were male. Fifty‐two patients with B‐ALL or NHL received humanised CD19 CAR T‐cell therapy, with a median of 3 (range, 1–8) previous lines of treatment and 17.19% had a history of haematopoietic stem cell transplantation. Twelve patients with SLE received Relmacabtagene autoleucel infusion. The overall incidence of CRS was 54.69%, with severe CRS (Grade 3–4) occurring in 3.13% of patients. ICANS was observed in 9.38% of patients. Tocilizumab and corticosteroids were administered during treatment in 2 and 29 patients, respectively (Supplementary table 1).

### Synchronous recovery of NK cells in peripheral blood and bone marrow after CD19 CAR T‐cell infusion

**Figure 1:**
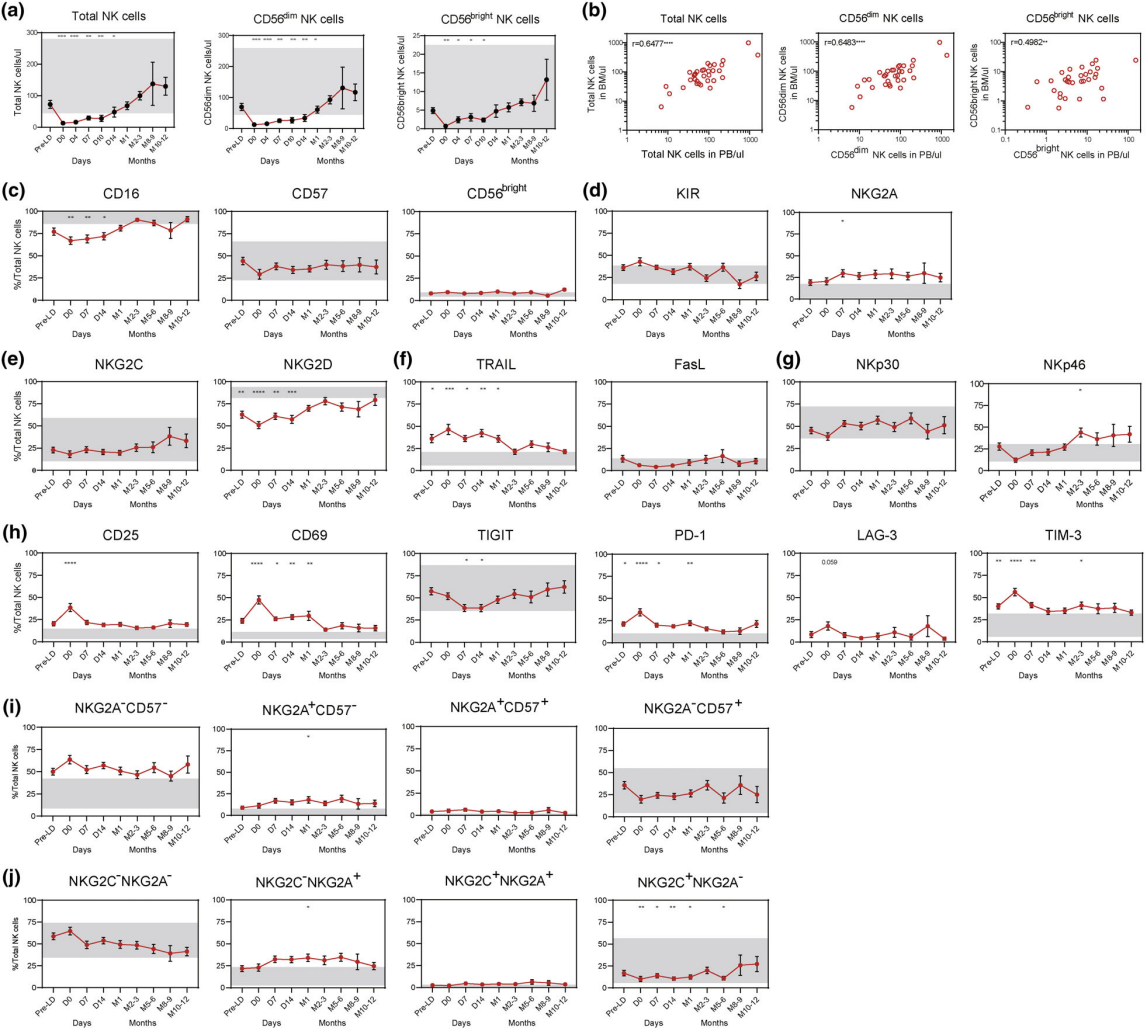
Recovery of NK‐cell number and receptor expression after CD19 CAR‐T cell therapy. **(a)** Dynamics of total, CD56^dim^, and CD56^bright^ NK‐cell counts after CD19 CAR T‐cell therapy. The grey interval represents the reference range (mean ± SD) from six healthy individuals. Black asterisks indicate statistically significant differences between the patient group and the normal reference range. **(b)** Correlation analysis of total, CD56^dim^, and CD56^bright^ NK cells between PB and BM. **(c–h)** Longitudinal expression of NK‐cell receptors associated with maturation **(c)**, inhibition **(d)**, cytotoxicity **(e–g)** and activation/exhaustion **(h)** from pre‐LD to one‐year post‐CTI. **(i)** Changes in NK‐cell subsets based on the expression of NKG2A and CD57. **(j)** Dynamic alterations in the NKG2C^−^NKG2A^−^, NKG2C^−^NKG2A^+^, NKG2C^+^NKG2A^+^ and NKG2C^+^NKG2A^−^ subpopulations. The grey interval represents the reference range (mean ± SD) from 10 healthy individuals **(c–j)**. Statistical analysis was performed using one‐way ANOVA with multiple comparisons **(a, c–j)** and Spearman correlation analysis **(b)**. *P < 0.05, **P < 0.01, ***P < 0.001, ****P < 0.0001. ANOVA, analysis of variance; BM, bone marrow; CAR, chimeric antigen receptor; CTI, CAR‐T cell infusion; D, day; LD, lymphodepletion; M, month; NK, natural killer; PB, peripheral blood; SD, standard deviation.

We performed a longitudinal analysis of patients receiving CD19 CAR T‐cell therapy to evaluate the recovery of CD3^−^CD56^+^ NK cells. The proliferation kinetics of total CD56^+^ NK cells and CD56^dim^ NK cells showed a consistent pattern: cell counts reached a nadir at Day 0 and gradually returned to the normal reference range by approximately one month post‐infusion (Figure 1a). In contrast, CD56^bright^ NK cells exhibited a narrower range of fluctuation, with significantly reduced counts between Day 0 and Day 10, and recovery to normal levels after Day 14 (Figure 1a).

Correlation analysis of paired peripheral blood (PB) and bone marrow (BM) samples from the same patients at corresponding time points revealed strong positive correlations in total, CD56^dim^, and CD56^bright^ NK‐cell counts, indicating a synchronous recovery pattern between PB and BM compartments (Figure 1b).

Given that NK‐cell reconstitution has been linked to IL‐15 levels in the context of haematopoietic stem cell transplantation, we further investigated this association in the setting of CAR T‐cell therapy. However, no significant correlation was observed between IL‐15 levels on Days 0, 3 or 4 and NK‐cell counts at one‐month post‐infusion (*r* = −0.0334, *P* = 0.8659; Supplementary figure 1a,b).

### Restoration of NK‐cell surface receptors after CD19 CAR T‐cell therapy

To evaluate the impact of CD19 CAR T‐cell therapy on the surface receptor profile of circulating NK cells, we conducted a longitudinal analysis from the LD chemotherapy phase through to one‐year post‐infusion. Among maturation‐related markers, CD16 expression significantly declined after LD but returned to normal levels by one month post‐CTI (Figure 1c). CD57 expression remained within the normal reference range throughout the study period (Figure 1c). Meanwhile, levels of the immaturity‐associated marker CD56^bright^ maintained stable at the upper limit of the reference range (Figure 1c). Moreover, the CD56^bright^/CD56^dim^ ratio also showed no significant difference (Supplementary figure 2a).

For inhibitory receptors, KIR expression remained relatively unchanged, whereas NKG2A levels were elevated at Day 7 following CAR‐T cell infusion (CTI, Figure 1d). For the NKG2 family receptors, NKG2C expression remained stable and within the normal range (Figure 1e). Notably, NKG2D expression was reduced prior to LD chemotherapy and remained suppressed until approximately 1 month after CTI (Figure 1e).

We also assessed TNF family members involved in NK cell‐mediated apoptosis. TRAIL, primarily expressed by immature NK cells, was elevated from the LD phase through to one‐month post‐infusion, while FasL, associated with mature NK cells, remained at normal levels (Figure 1f).^13^

Natural cytotoxicity receptors NKp30 and NKp46 are typically expressed on resting NK cells.^14^ In our study, their expression remained largely unchanged, with only a modest elevation of NKp46 observed at the 2–3‐month time point (Figure 1g).

Chronic immune activation is often accompanied by increased expression of activation and exhaustion markers on NK cells.^15^ In this cohort, NK cells displayed strong activation features, including elevated CD25 and CD69 expression, along with upregulation of exhaustion markers PD‐1, LAG‐3, and TIM‐3, most notably at Day 0 (Figure 1h).

In addition, we analysed the subsets of NK cells. Subpopulations defined by NKG2A and CD57 expression did not show any statistically significant differences compared to the normal reference range (Figure 1i). In contrast, among the subsets defined by NKG2C and NKG2A, the proportion of NKG2C^+^NKG2A^−^ NK cells was significantly reduced during the period from day 0 to one‐month post‐infusion (Figure 1j). The adaptive NK cell subset, characterised as CD56^dim^NKG2C^+^CD57^+^ NK cells, did not show marked changes during the NK cell reconstitution phase following CAR‐T cell therapy (Supplementary figure 2b).

These findings suggest that during NK‐cell reconstitution following CAR T‐cell therapy, cytotoxic and functional receptor expression is significantly modulated, with most receptors normalising after the first month. Additionally, NK cells display a pronounced activation phenotype early after CTI.

### Similar patterns of NK cell recovery in cancer and SLE patients treated with CD19 CAR‐T cells

Different disease contexts may influence NK cell reconstitution. We further analysed the dynamic changes in NK cell numbers and phenotypes between cancer patients (including NHL and B‐ALL) and SLE patients following CD19 CAR‐T cell therapy. The results showed that both total NK cell counts and receptor phenotypes exhibited similar recovery trends in the two groups (Supplementary figure 3a,b).

The only notable difference was a significantly lower expression of NKG2C in SLE patients from prior to LD (pre‐LD) to months 2–3 post‐CTI, compared to the normal reference range (Supplementary figure 3b). In addition, although not statistically significant, CD57 expression also tended to be lower in the SLE group. We speculate that SLE patients may have a lower proportion of adaptive NK cells (defined as CD56^dim^NKG2C^+^CD57^+^), which are typically associated with prior CMV infection.^16^ As shown in Supplementary figure 3c, the frequency of adaptive NK cells was indeed lower in SLE patients compared to the cancer group (NHL and B‐ALL).

These results suggest that, under the context of CD19 CAR‐T cell therapy, NK cell numeric and phenotypic recovery follows a similar trend in both cancer and SLE patients, despite minor disease‐specific differences.

### Impaired numerical recovery of NK cells in the CRS group

Cytokine storm during CAR T‐cell treatment has been shown to impair haematopoietic recovery, and we hypothesised that CRS may similarly affect NK‐cell reconstitution.^17^ To investigate the impact of CRS on NK‐cell recovery, patients were stratified into two groups based on the occurrence of CRS.

**Figure 2:**
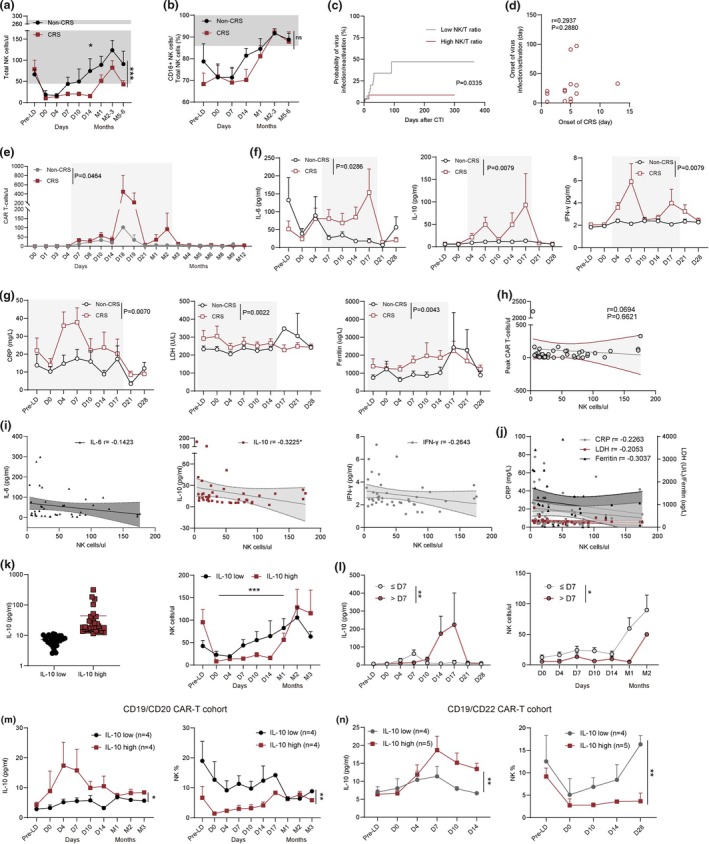
High IL‐10 levels during CRS are associated with delayed NK‐cell recovery. **(a)** Dynamics of NK‐cell count recovery in CRS and non‐CRS groups. **(b)** Dynamics of CD16 expression on NK cells in CRS and non‐CRS groups. **(c)** Virus infection/reactivation rates in the high (≥ median) NK/T ratio and low (< median) NK/T ratio groups at M1 time point. **(d)** Correlation between the timing of viral infection/reactivation and the timing of CRS onset. **(e–g)** Monitoring of CAR T‐cell expansion **(e)**, inflammatory cytokine levels **(f)**, and inflammatory protein levels **(g)** in CRS and non‐CRS groups. **(h–j)** Correlation analysis between NK‐cell counts and peak CAR T‐cell expansion **(h)**, inflammatory cytokines **(i),** and inflammatory proteins **(j)**. Cytokine and protein levels used for correlation analyses were: average levels of IL‐6 (Days 7–17), IL‐10 (Days 4–17), IFN‐γ (Days 4–17), CRP (pre‐LD to Day 17), LDH (pre‐LD to Day 14), and ferritin (pre‐LD to Day 14). **(k)** IL‐10 levels and NK‐cell recovery dynamics in high (≥ median) vs. low IL‐10 groups. **(l)** IL‐10 levels and NK‐cell recovery in CRS patients grouped by IL‐10 peak occurring on or before Day 7 (≤ D7) vs. after Day 7 (> D7). **(m, n)** Validation in CD19/CD20 CAR‐T and CD19/CD22 CAR‐T cohorts showing IL‐10 levels and NK‐cell frequency in high (≥ median) vs. low IL‐10 groups. Statistical analysis was performed using two‐way ANOVA **(a, b, k–n)**, Mann–Whitney *U*‐test **(c–g)**, and Spearman correlation analysis **(h–j)**. *P < 0.05, **P < 0.01, ***P < 0.001, ****P < 0.0001, ns, not significant. ANOVA, analysis of variance; CRP, C‐reactive protein; CRS, cytokine release syndrome; CTI, CAR T‐cell infusion; D, day; IFN, interferon; IL, interleukin; LD, lymphodepletion; LDH, lactate dehydrogenase; M, month; NK, natural killer.

In the CRS group, NK‐cell counts exhibited a markedly delayed recovery, becoming evident around Day 14, whereas in the non‐CRS group, recovery was observed as early as Day 4 (Figure 2a). The recovery of CD16 expression was also relatively delayed in the CRS group, although the difference was not statistically significant (Figure 2b). Poor NK cell recovery may increase the risk of viral infections. In our cohort, a total of 14 patients experienced 18 viral infection or reactivation events following CAR‐T cell therapy. These included infections with CMV, Epstein–Barr virus, Herpes simplex virus, influenza virus, and SARS‐CoV‐2. We observed a higher probability of viral infections/reactivation in the low NK/T ratio (defined at month 1 time point) group (hazard ratio = 4.512, Figure 2c). In addition, there was a weak positive correlation between the timing of CRS onset and the occurrence of viral infections (Figure 2d). Notably, the recovery kinetics of other NK cell markers showed similar trends between the two groups (Supplementary figure 4).

### IL‐10 levels are negatively associated with NK cell recovery

Given the pronounced delay in NK‐cell count recovery observed in the CRS group, we further explored potential factors influencing NK‐cell reconstitution. Consistent with a previous report, patients with CRS exhibited significant CAR T‐cell expansion and elevated levels of cytokines (IL‐6, IL‐10, and interferon‐γ [IFN‐γ]), inflammatory markers (C‐reactive protein [CRP] and ferritin) and lactic dehydrogenase (LDH), while no significant differences were observed in IL‐2, IL‐4, or tumor necrosis factor‐α (TNF‐α) levels (Figure 2e–g and Supplementary figure 5).^18^

Correlation analysis revealed a negative association between the anti‐inflammatory cytokine IL‐10 and NK cell counts, whereas other factors showed no significant correlation (Figure 2h–j), suggesting a potential link between IL‐10 levels and NK‐cell recovery. To further investigate this, we stratified patients based on IL‐10 concentrations. Patients in the high (≥ median) IL‐10 group had significantly lower NK‐cell counts during the day 0–month 1 period compared to those with low IL‐10 levels (Figure 2k). Since patients in the CRS group exhibited two distinct IL‐10 peaks, we further divided them based on the timing of IL‐10 peak appearance (peak ≤ day 7 or > day 7). Notably, patients whose IL‐10 peaks occurred later also exhibited delayed NK‐cell recovery (Figure 2l). To validate these findings, we analysed two additional clinical trial cohorts from our centre—CD19/CD20 CAR‐T and CD19/CD22 CAR‐T—and both cohorts consistently demonstrated lower NK cell proportions in the high IL‐10 group (Figure 2m and n).

Collectively, these results indicate that elevated IL‐10 levels in patients with CRS are associated with delayed NK cell recovery.

### IL‐10 promotes apoptosis and ferroptosis of NK cells

**Figure 3:**
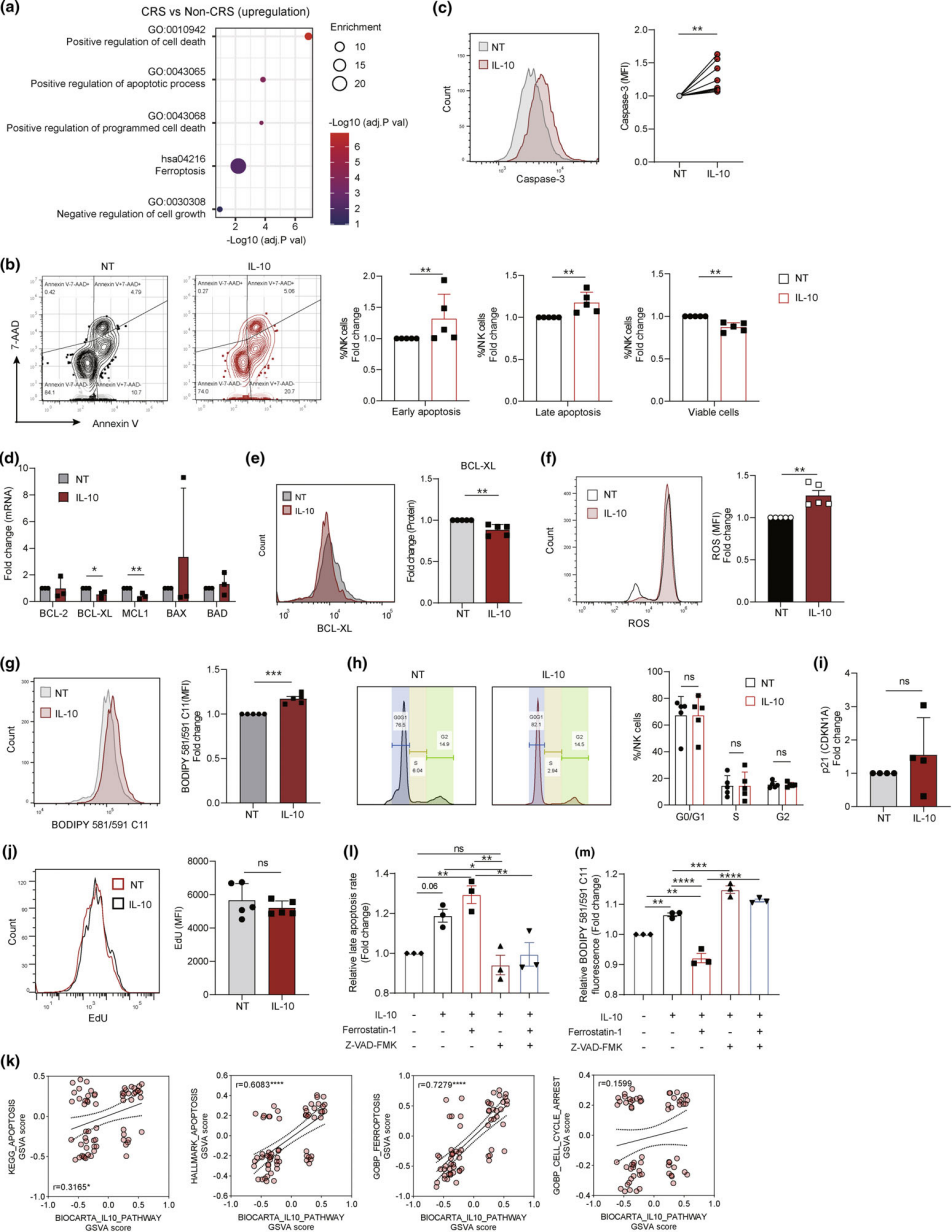
IL‐10 promotes NK‐cell apoptosis and ferroptosis. **(a)** Enriched pathways in plasma proteomic profiles of CRS vs. non‐CRS patients treated with huCD19 CAR‐T cells. **(b, c)** Effects of IL‐10 on NK‐cell apoptosis and caspase‐3 (*n* = 5, two biological replicates) expression. **(d)** RT‐qPCR analysis of IL‐10‐induced expression changes in key pro‐ and anti‐apoptotic genes in NK cells. **(e)** BCL‐XL levels in the NT or IL‐10 treated group detected by flow cytometry. **(f, g)** Effects of IL‐10 on NK‐cell ROS levels **(f)** and lipid peroxidation **(g)**. **(h)** Impact of IL‐10 on NK‐cell cell cycle distribution. **(i)** RT‐qPCR analysis of cell cycle inhibitor p21 (CDKN1A) expression after IL‐10 treatment. **(j)** Representative flow cytometry plots (left) and quantification (right) of EdU incorporation in IL‐10‐treated NK cells. **(k)** Correlation analysis between GSVA scores of the BIOCARTA_IL10_PATHWAY and scores for KEGG_APOPTOSIS, HALLMARK_APOPTOSIS, GOBP_FERROPTOSIS, and GOBP_CELL_CYCLE_ARREST based on RNA sequencing data of NK cells. **(l, m)** Effects of ferrostatin‐1 and/or Z‐VAD‐FMK on apoptosis **(l)** and lipid peroxidation **(m)** in NK cells in rescue experiments. Statistical analysis was performed using two‐way ANOVA with multiple comparisons **(h, l, m)**, unpaired Student's *t* test **(b–g, i, j)**, and Pearson correlation analysis **(k)**. *P < 0.05, **P < 0.01, ***P < 0.001, ****P < 0.0001, ns, not significant. ANOVA, analysis of variance; CRS, cytokine release syndrome; IL, interleukin; NK, natural killer; NT, non‐treated condition; ROS, reactive oxygen species; RT‐qPCR, real‐time quantitative polymerase chain reaction; GSVA, gene set variation analysis.

Proteomic analysis of plasma from patients treated with huCD19 CAR‐T cells revealed that, compared to the non‐CRS group, the CRS group showed enrichment of pathways related to the positive regulation of apoptosis and ferroptosis, as well as the negative regulation of cell growth (Figure 3a).^19^ In addition, IL‐10 exerts dual effects on NK cells: on one hand, it can enhance NK cell cytotoxicity by upregulating granzyme B expression (Supplementary figure 6), while on the other hand, it promotes the expression of genes involved in cell cycle arrest and apoptosis in NK cells.^20^

To further investigate the mechanism by which IL‐10 affects NK‐cell recovery, we conducted *in vitro* experiments using NK cells derived from healthy donors. Cells were cultured in the presence or absence of IL‐10 and assessed for apoptosis, ferroptosis, cell cycle, and proliferation. IL‐10 treatment increased the proportion of early apoptotic and late apoptotic cells and reduced the percentage of viable NK cells (Figure 3b). These were accompanied by elevated expression of caspase‐3 (Figure 3c), reduced levels of anti‐apoptotic molecules BCL‐XL (Figure 3d and e) and reduced MCL1 expression at the mRNA level (Figure 3d). IL‐10‐treated NK cells also exhibited increased levels of reactive oxygen species (ROS) and lipid peroxidation (Figure 3f and g). However, IL‐10 had no significant impact on the cell cycle, gene expression of the cell cycle inhibitor p21 (CDKN1A) or EdU incorporation (Figure 3h–j). Furthermore, the increase in apoptosis‐ and ferroptosis‐related markers was also validated in patient‐derived NK cells in the presence of IL‐10 (Supplementary figure 7).

Analysis of publicly available RNA sequencing data (GSE269629) showed that the gene set variation analysis score of BIOCARTA_IL10_PATHWAY in NK cells was positively correlated with apoptosis and ferroptosis signatures, but not with the cell cycle arrest signature (Figure 3k). Additionally, Z‐VAD‐FMK reduced the IL‐10‐mediated increase in late apoptotic NK cells, while ferrostatin‐1 attenuated the IL‐10‐induced elevation of lipid peroxidation (Figure 3l and m), further implicating IL‐10 signaling in the apoptotic and ferroptotic processes of NK cells.

## Discussion

CAR T‐cell therapy has demonstrated potent anti‐tumor activity and can effectively eliminate target cells, leading to meaningful clinical responses.^21^ However, the combined effects of LD chemotherapy (typically fludarabine and cyclophosphamide) and CTI significantly alter patients' immune‐cell composition and impair immune reconstitution. To date, studies on immune recovery have primarily focussed on T cells,^21^ B cells^22^, and neutrophils.^23^ In contrast, the dynamics of NK cells—key effectors in antiviral and anti‐tumor immunity—remain poorly characterised following CAR‐T cell therapy. In this study, we analysed both NK‐cell immunophenotype and clinical data from two independent CD19 CAR‐T cell clinical trials to comprehensively assess NK‐cell reconstitution and remodelling. Our aim was to identify key features, associated factors, and potential clinical implications of NK‐cell recovery in this therapeutic context.

This study was based on longitudinal monitoring of dynamic changes in both the number and phenotype of NK cells. NK cell counts reached their nadir at Day 0, followed by a steady increase over time. Notably, the proliferation of PB‐NK and BM‐NK exhibited synchronised trends. These findings suggest that the recovery of PB‐NK cells is primarily driven by *de novo* generation from haematopoietic precursors, rather than homeostatic proliferation in response to lymphopenia or cytokine‐induced expansion.^24^

During CRS, NK cell recovery was delayed, likely because of the massive expansion of CAR‐T cells, elevated levels of inflammatory cytokines and increased LDH levels.^25^ In this study, we observed a negative correlation between IL‐10 concentration and NK‐cell counts—an association that has also been supported by recently published findings.^5^ As an anti‐inflammatory cytokine, IL‐10 exerts dual regulatory effects on immune cells. On one hand, IL‐10 can promote CAR‐T cell proliferation and effector functions, and induce stem‐like memory responses in lymphoid organs, thereby contributing to long‐term tumor control.^26^ On the other hand, IL‐10 has been shown to suppress the expansion of virus‐specific CD4^+^ and CD8^+^ T cells during SARS‐CoV‐2 infection, and to drive systemic T‐cell dysfunction via IFN‐induced IL‐10 production in the context of chronic liver injury.^27^, ^28^ Regarding NK cells, IL‐10 can enhance their effector functions through mTORC1‐mediated metabolic reprogramming, but has also been reported to suppress NK‐cell activation during bacterial infections.^29^, ^30^ In chronic hepatitis B virus infection, IL‐10 blockade has been shown to restore NK‐cell IFN‐γ production, thereby enhancing their non‐cytolytic antiviral functions.^31^ These findings suggest that IL‐10 plays context‐dependent roles in immune regulation across different pathological conditions. In addition, our previous study demonstrated that IL‐10, in combination with the endothelial activation and stress index, could predict bleeding events following CD19 CAR‐T therapy.^32^ In the present study, elevated IL‐10 levels during CRS were associated with delayed NK cell recovery, potentially through the delivery of pro‐apoptotic and pro‐ferroptotic signals to NK cells. Moreover, IL‐10 may also indirectly suppress NK cell activation by inhibiting IL‐12 production from dendritic cells—a mechanism previously proposed in other studies, but requiring further investigation in the context of CRS.^33^

Natural killer‐cell deficiency and impaired reconstitution have been associated with an increased risk of viral infections and poor clinical outcomes.^5^, ^11^, ^34^ Therefore, for patients exhibiting delayed NK‐cell recovery after CAR‐T cell therapy, strategies to enhance NK‐cell expansion and function warrant further exploration. Potential approaches may include autologous NK‐cell infusion alone or in combination with cytokines, CD16‐binding antibodies or immune engagers.^35^, ^36^

Our findings also carry additional clinical implications. For example, ongoing clinical trials are investigating the use of CD19 CAR‐T cells in combination with CD20 mAbs for B‐cell NHL (e.g. NCT04002401 and NCT04889716). The dynamic expression pattern of CD16 observed after CD19 CAR‐T cell therapy in our study provides a biological rationale supporting the design of such combination strategies.

This study has several limitations. Our analysis included patients from two clinical trials, and NK‐cell recovery characteristics may vary depending on the specific CD19 CAR‐T cell product used. However, since both products incorporated the 4‐1BB costimulatory domain and utilised an identical LD regimen, we consider the impact of product variability on NK‐cell recovery to be minimal. Our study reveals a potential mechanism by which IL‐10 regulates NK cell recovery. More complex regulatory pathways may be further explored in future, for instance, through single‐cell sequencing of patient samples. In addition, although most experiments in this study were conducted using NK cells from healthy donors, we subsequently validated the findings in patient‐derived NK cells, and it seems that the conclusions from both sources are consistent.

In summary, this study is the first to comprehensively characterise the recovery dynamics of circulating NK cells following CD19 CAR‐T cell therapy and to identify associated influencing factors. Our findings highlight the clinical importance of NK‐cell reconstitution in this setting. For patients undergoing CAR‐T cell therapy, particularly those with impaired NK‐cell recovery, strategies aimed at promoting NK‐cell expansion and function, as well as preventing viral infection/reactivation and disease relapse/progression, are warranted and merit further investigation.

## Conclusions

In this study, we comprehensively characterised the dynamic reconstitution of NK‐cell counts and phenotypic profiles following CAR‐T cell therapy and highlighted a previously underappreciated role of IL‐10 in shaping NK‐cell recovery, particularly in the context of CRS.

## Methods

Details regarding the quantification of plasma IL‐15, cell cycle assay, EdU incorporation assay, real‐time quantitative‐polymerase chain reaction (RT‐qPCR), and sequencing data analysis are provided in the Supplementary materials.

### Patients and sample collection

This study enrolled 64 patients who received CD19 CAR T‐cell treatment at Wuhan Union Hospital, Huazhong University of Science and Technology, Wuhan, China. Of these, 52 patients—diagnosed with either NHL or B‐ALL—were treated with humanised CD19 CAR T‐cell products (NCT04008251). Patients in the cohort received LD chemotherapy (fludarabine 30 mg/m^2^ and cyclophosphamide 250 mg/m^2^) on Days ‐5– ‐3, followed by infusion of CAR^+^ T‐cells at 1 × 10^6^ cells/kg on Days 0 and 1.^32^ An additional 12 patients with SLE were enrolled in a separate trial (NCT05765006) to receive Relmacabtagene autoleucel. These patients underwent a LD regimen consisting of fludarabine (30 mg/m^2^) and cyclophosphamide (250 mg/m^2^) administered over three consecutive days, completed within 2–7 days prior to CTI. The CAR T‐cell products used in both trials incorporated a second‐generation CAR structure, containing a 4‐1BB costimulatory domain and a CD3ζ signal peptide. All patients signed informed consent in accordance with the Declaration of Helsinki.

PB samples were collected at multiple time points: pre‐LD, and on Days 0, 4, 7, 10 and 14, as well as Months 1, 2–3, 5–6, 8–9 and 10–12 post‐CTI. BM samples were collected from NHL and B‐ALL patients at baseline (pre‐LD) or during follow‐up visits. In addition, PB samples from 10 healthy individuals were obtained as controls.

### Flow cytometry

The antibodies and viability dyes used in this study are listed in the Supplementary table 2. Absolute quantification of total NK cells, CD56^dim^ NK cells, CD56^bright^ NK cells, and CAR T‐cells was performed using fresh samples and counting beads (BioLegend, San Diego, CA, USA), following previously established protocols.^37^

Phenotypic analysis of NK cells was conducted by isolating peripheral blood mononuclear cells (PBMCs) via density gradient centrifugation, followed by surface staining with specific antibodies at 4°C for 30 min. For the detailed gating strategy, please refer to Supplementary figure 8. Flow cytometry was performed using a BD LSRFortessa X‐20 cytometer (BD Biosciences, San Jose, CA, USA) and a BD FACSVia system (BD Biosciences, San Jose, CA, USA), and data were analysed using the FlowJo software (version 10.8.1; FlowJo LLC, Ashland, OR, USA).

### Clinical data collection

Clinical data, including patient demographics and routine laboratory measurements—such as cytokine levels (IL‐2, IL‐4, IL‐6, IL‐10, IFN‐γ, and TNF‐α), inflammatory markers (CRP and ferritin), and LDH—were collected from the Electronic Medical Record System. The assessment and grading of CRS and ICANS were conducted in accordance with the American Society for Transplantation and Cellular Therapy guidelines.^38^ The criteria used to define viral infection events were consistent with previous studies.^39^ Briefly, both clinically diagnosed and microbiologically confirmed infections were included in the analysis. Respiratory viruses were defined by any of the following: positive viral culture or polymerase chain reaction (PCR) from nasopharyngeal swabs or sputum; detection of virus in bronchoalveolar lavage fluid; or presence of prominent respiratory symptoms with imaging showing new or progressive pulmonary infiltrates after exclusion of bacterial infections. Herpes simplex virus infection is typically diagnosed based on characteristic skin or mucosal lesions, such as vesicles and ulcers, in combination with laboratory tests, including PCR and serologic assays. Other viruses, such as Epstein–Barr virus and CMV, were identified by positive findings in blood samples.

### NK cell isolation and culture

Peripheral blood mononuclear cells were isolated from healthy donors or patients using density gradient centrifugation. NK cells were enriched from PBMCs using the NK Cell Isolation Kit (Miltenyi Biotec, Bergisch Gladbach, Germany) according to the manufacturer's instructions.

For *in vitro* expansion, NK cells were cultured using the IL‐21 NK Cell Expansion Kit (Lifeark, Hangzhou Zhongying Biotechnology, Hangzhou, China) in NK cell culture medium (Lifeark, Hangzhou Zhongying Biotechnology, Hangzhou, China) supplemented with 200 U/mL recombinant human IL‐2 (PeproTech, Rocky Hill, NJ, USA). On Day 7 of culture, recombinant human IL‐21 (PeproTech, Rocky Hill, NJ, USA) was added at a final concentration of 30 ng/mL to promote further expansion and activation. The purity of NK cells was assessed by flow cytometry prior to subsequent experiments. Mycoplasma testing was not performed on the NK cells used in this study.

### Apoptosis assay and caspase‐3 detection

Natural killer cells were treated with or without 50 ng/mL IL‐10 (GenScript, Piscataway, NJ, USA) for 48 h,^20^, ^29^ followed by apoptosis analysis using the Annexin V/7‐AAD staining method (Apoptosis Detection Kit, Vazyme, Nanjing, China). Caspase‐3 activity was assessed using the GreenNuc™ Live Cell Caspase‐3 Activity Detection Kit (Beyotime Biotechnology, Shanghai, China). After staining according to the manufacturer's instructions, samples were analysed by flow cytometry.

### Reactive oxygen species and lipid peroxidation assays

Natural killer cells were treated with or without 50 ng/mL IL‐10 for 48 h.^20^, ^29^ Subsequently, intracellular ROS levels were measured using a ROS detection kit (Beyotime Biotechnology, Shanghai, China), and lipid peroxidation was assessed using the BODIPY 581/591 C11 probe (Beyotime Biotechnology, Shanghai, China), following the manufacturers' protocols.

### Rescue experiments

For rescue experiments, NK cells were pretreated with 2 μM ferrostatin‐1 (TargetMol, Shanghai, China) and/or 30 μM Z‐VAD‐FMK (Beyotime Biotechnology, Shanghai, China) for 0.5 h, followed by stimulation with 50 ng/mL IL‐10. After 48 h, these cells were collected for assessment of apoptosis and lipid peroxidation.

### Statistical analyses

Statistical comparisons were performed using the Mann–Whitney *U*‐test, unpaired Student's *t*‐test, one‐way analysis of variance (ANOVA) with multiple comparisons, and two‐way ANOVA with or without multiple comparisons. Correlation analysis was performed using Spearman or Pearson correlation analysis. Viral infection rates were compared using the log‐rank (Mantel‐Cox) test between two groups. Unless otherwise specified, data are presented as mean ± standard error of the mean (SEM). Normal reference ranges are shown as mean ± standard deviation (SD) based on healthy volunteers. All statistical tests were two‐sided, and *P* < 0.05 was considered statistically significant (*P < 0.05, **P < 0.01, ***P < 0.001, ****P < 0.0001; ns, not significant). Data were analysed and visualised using GraphPad Prism (version 8.0.2; GraphPad Software, Boston, MA) and R software (version 4.1.2; R Foundation for Statistical Computing, Vienna, Austria).

In accordance with the journal's policy on the use of generative AI, ChatGPT (OpenAI) was used for language editing purposes only. No AI tools were used in data analysis or interpretation. The authors are fully responsible for the content of this manuscript.

## Author contributions

**Xindi Wang:** Conceptualization; investigation; funding acquisition; writing – original draft; methodology; visualization; writing – review and editing; validation; formal analysis; data curation; software. **Zhihan Chen:** Investigation. **Chenggong Li:** Investigation. **Lu Tang:** Methodology. **Heng Mei:** Writing – review and editing; project administration; resources; funding acquisition; conceptualization; supervision. **Jianghua Wu:** Methodology; data curation. **Yingying Li:** Investigation; methodology; data curation; validation. **Wenjing Luo:** Writing – review and editing; conceptualization; methodology. **Yu Hu:** Resources; writing – review and editing; project administration; conceptualization; supervision. **Qiaolin Liu:** Investigation.

## Conflict of interest

The authors declare no conflict of interest.

## Ethics approval

This study was approved by the medical ethics committee of Wuhan Union Hospital, Tongji Medical College, Huazhong University of Science and Technology, Wuhan, China, [2023] 0754‐01 and [2022] 0074‐02. Participants gave informed consent to participate in the study.

## Funding

This work was supported by grants from the National Natural Science Foundation of China (82425003, 82330005, and 82350103), the National Key R&D Program of China (No. 2022YFC2304600), the Fundamental Research Funds for the Central Universities (YCJJ20252118) and the Wuhan Science and Technology Achievement Transformation Project (2024030803010193).

## Consent for publication

Not applicable.

## Supporting information


Supplementary table 1
Supplementary table 2
Supplementary table 3
Supplementary figure 1
Supplementary figure 2
Supplementary figure 3
Supplementary figure 4
Supplementary figure 5
Supplementary figure 6
Supplementary figure 7
Supplementary figure 8

## Data Availability

All data generated or analysed during this study are included in this published article and its Supplementary materials.
