# Supplementary material for: High IL‐10 levels during CRS are negatively associated with NK‐cell recovery after CAR‐T cell therapy
Source: Clin Transl Immunology. 2026 Jul 24;15(7):e70116. doi: 10.1002/cti2.70116 (PMC13396887; doi:10.1002/cti2.70116)
Supplement: Supplementary file 1 — Supplementary table 1 Supplementary table 2 Supplementary table 3 Supplementary figure 1 Supplementary figure 2 Supplementary figure 3 Supplementary figure 4 Supplementary figure 5 Supplementary figure 6 Supplementary figure 7 Supplementary figure 8 [file CTI2-15-e70116-s001.docx]

**Supplemental materials for**

**High IL-10 levels during CRS are negatively associated with NK-cell recovery after CAR-T cell therapy**

**Xindi Wang,^1,2,3*^ Wenjing Luo****,^1,2,3*^ Yingying Li,^1,2,3*^ Jianghua Wu,^1,2,3^ Lu Tang,^1,2,3^ Chenggong Li,^1,2,3^ Qiaolin Liu,^1,2,3^ Zhihan Chen,^1,2,3^ Yu Hu^1,2,3#^, and Heng Mei,^1,2,3#^**

1. Institute of Hematology, Union Hospital, Tongji Medical College, Huazhong University of Science and Technology, Wuhan, 430022, China.
2. Hubei Clinical Medical Center of Cell Therapy for Neoplastic Disease, Wuhan, 430022, China.
3. Key Laboratory of Biological Targeted Therapy (Huazhong University of Science and Technology), Ministry of Education, Wuhan, 430022, China.

*****XW, WL, and YL contributed equally as co-first authors.

**^#^**HM and YH contributed equally as co-senior authors.

The PDF file includes:

Supplementary Tables 1 to 3

Supplementary Figures 1 to 8

**Methods**

**Quantification of plasma IL-15**

Plasma IL-15 levels of patients at different time points were detected by a human IL-15 ELISA kit from Proteintech (KE00102, Rosemont, IL, USA).

**Cell cycle assay**

NK cells were treated with or without 50 ng/mL IL-10 for 48 hours. Cell cycle distribution was assessed using the Cell Cycle and Apoptosis Analysis Kit (Beyotime Biotechnology, Shanghai, China) according to the manufacturer’s instructions.

**EdU incorporation assay**

NK cells were treated with or without 50 ng/mL IL-10 for 48 hours, followed by a 2-hour incubation with EdU. EdU incorporation was then assessed using the EdU Cell Proliferation Detection Kit (Beyotime Biotechnology, Shanghai, China) according to the manufacturer’s instructions.

**RT-qPCR**

Total RNA was extracted using RNA extraction kits (CWBio, Beijing, China), followed by genomic DNA removal and reverse transcription into cDNA using the HiScript III 1st Strand cDNA Synthesis Kit (Vazyme, Nanjing, China). RT-qPCR was performed using ChamQ SYBR qPCR Master Mix (Vazyme, Nanjing, China) according to the manufacturer’s instructions. mRNA expression levels were normalized to ACTB (β-actin) as the internal control. Primer sequences for target genes are provided in Supplemental table 3.

**Sequencing data analysis**

Plasma proteomics sequencing data were obtained from Wu J, et al., previously published by our laboratory.^1^ As previously described, differentially expressed proteins were defined by a p-value < 0.05 and an absolute log₂ fold change > 0.585. Pathway enrichment analysis of the identified proteins was performed using the Metascape web-based platform, which integrates Gene Ontology (GO) biological processes, Kyoto Encyclopedia of Genes and Genomes (KEGG) pathways, and Hallmark gene sets.^2^ Gene expression data from the GEO database (GSE269629)^3^ were analyzed using Gene Set Variation Analysis (GSVA) with the GSVA R package to calculate activity scores for specific pathways.

**Supplemental table 1. Patient characteristics**

| Characteristics | Total (n=64) | huCD19 CAR T-cell (n=52) | Relmacabtagene autoleucel (n=12) |
| --- | --- | --- | --- |
| Age, yr, median (range) | 46 (18-80) | 50 (18-80) | 27.5 (19-37) |
| Males (%) | 34 (53.13) | 33 (63.46) | 1 (8.33) |
| Diagnosis, n (%) |  |  |  |
| B-ALL | 19 (29.69) | 19(36.54) | / |
| NHL | 33 (51.56) | 33 (63.46) | / |
| SLE | 12 (18.75) | / | 12 (100.00) |
| Prior lines of therapy, median (range) | 3 (1-8) | 3 (1-8) | / |
| Prior transplant, n (%) | 11 (17.19) | 11 (21.15) | / |
| CRS, n (%) |  |  |  |
| Grade 0 | 29 (45.31) | 26 (50.00) | 3 (25.00) |
| Grade 1-2 | 33 (51.56) | 24 (46.15) | 9 (75.00) |
| Grade 3-4 | 2 (3.13) | 2 (3.85) | 0 |
| ICANS, n (%) |  |  |  |
| Grade 0 | 58 (90.63) | 46 (88.46) | 12 (100.00) |
| Grade 1-2 | 3 (4.69) | 3 (5.77) | 0 |
| Grade 3-4 | 3 (4.69) | 3 (5.77) | 0 |
| Treatments, n (%) |  |  |  |
| Use of tocilizumab | 2 (3.13) | 2 (3.85) | 0 |
| Use of steroids | 29 (45.31) | 16 (30.77) | 7 (58.33) |

B-ALL, B-cell acute lymphoblastic leukemia; NHL, non-Hodgkin lymphoma; SLE, systemic lupus erythematosus; CRS, cytokine release syndrome; ICANS, immune effector cell-associated neurotoxicity syndrome.

**Supplementary table 2. Antibodies used in this study**

| **Antibodies** | **Source** | **Catalogue** | **Clone** |
| --- | --- | --- | --- |
| Zombie Red Fixable Viability Kit | BioLegend | 423109 | / |
| BD Horizon™ Fixable Viability Stain 620 | BD Biosciences | 564996 | / |
| Anti-human CD56 BV606 | BioLegend | 362538 | 5.1H11 |
| Anti-human CD56 PE | BioLegend | 304606 | MEM-188 |
| Anti-human CD3 PerCP/Cyanine5.5 | BioLegend | 317336 | OKT3 |
| Anti-human CD16 BV421 | BD Biosciences | 562874 | 3G8 |
| Anti-human NKG2D PE/Cyanine7 | BioLegend | 320812 | 1D11 |
| Anti-human NKP46 BV510 | BioLegend | 331924 | 9E2 |
| Anti-human NKP30 APC | BioLegend | 325212 | P30-15 |
| Anti-human KIR3DL1 AF700 | BioLegend | 312712 | DX9 |
| Anti-human FasL PE | BioLegend | 306407 | NOK-1 |
| Anti-human PD1 BV605 | BioLegend | 329924 | EH12.2H7 |
| Anti-human LAG3 BV510 | BioLegend | 369318 | 11C3C65 |
| Anti-human TIM3 APC | BioLegend | 345012 | F38-2E2 |
| Anti-human TIGIT BV421 | BioLegend | 372710 | A15153G |
| Anti-human CD25 PE/Cyanine7 | BioLegend | 302612 | BC96 |
| Anti-human CD69 APC/Cyanine7 | BioLegend | 310914 | FN50 |
| Anti-human CD57 PE/Cyanine7 | BioLegend | 359624 | HNK-1 |
| Anti-human NKG2A AF488 | BioLegend | 375124 | S19004C |
| Anti-human NKG2C PE | BioLegend | 375003 | S19005E |
| Anti-human TRAIL APC | BioLegend | 308209 | RIK-2 |
| BCL-XL rabbit monoclonal antibody | Beyotime Biotechnology | AB126 | / |
| AF488 goat anti-rabbit IgG (H+L) | Beyotime Biotechnology | A0423 | / |

**Supplemental table 3. List of primer sequences for RT-qPCR used in the study**

| **Oligonucleotides** | **Sequences** |
| --- | --- |
| ACTB forward primer | TCCTGTGGCATCCACGAAACT |
| ACTB reverse primer | GAAGCATTTGCGGTGGACGAT |
| BCL-2 forward primer | GAGGATTGTGGCCTTCTTTG |
| BCL-2 reverse primer | GCCGGTTCAGGTACTCAGTC |
| BCL-XL forward primer | CTGAATCGGAGATGGAGACC |
| BCL-XL reverse primer | TGGGATGTCAGGTCACTGAA |
| MCL1 forward primer | AAACTGGGGCAGGATTGTGA |
| MCL1 reverse primer | CCAGTCCCGTTTTGTCCTTA |
| BAD forward primer | TCCCAGAGTTTGAGCCGAGT |
| BAD reverse primer | ATGTGGAGCGAAGGTCACTG |
| BAX forward primer | CAGCTCTGAGCAGATCATGAAGACA |
| BAX reverse primer | GCCCATCTTCTTCCAGATGGTGAGC |
| CDKN1A forward primer | GCAGACCAGCATGACAGATTT |
| CDKN1A reverse primer | GGATTAGGGCTTCCTCTTGGA |

**Supplemental figure 1**


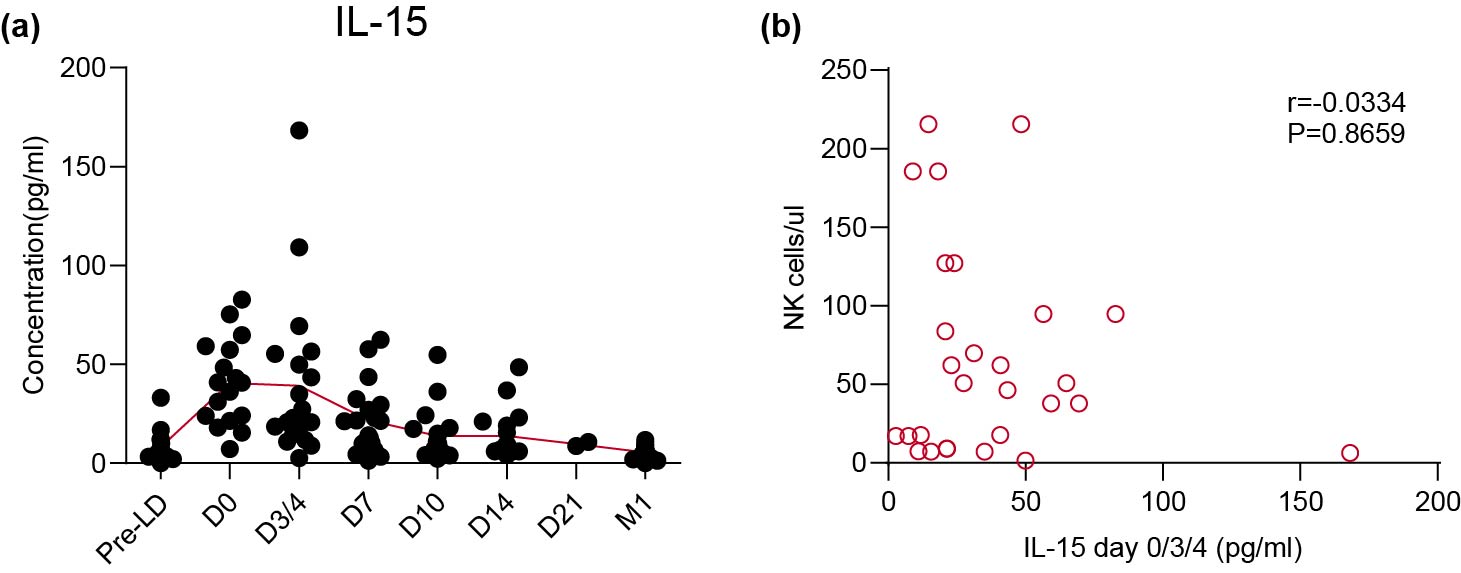


Supplemental figure 1. IL-15 levels following CTI and correlation with NK-cell counts.

**(a)** Plasma IL-15 levels within one month after CTI. **(b)** Correlations between IL-15 levels on days 0, 3, and 4 and NK-cell counts at month 1.

IL, interleukin; LD, lymphodepletion; D, day; M, month; NK, natural killer; CTI, CAR T-cell infusion.

**Supplemental figure 2**


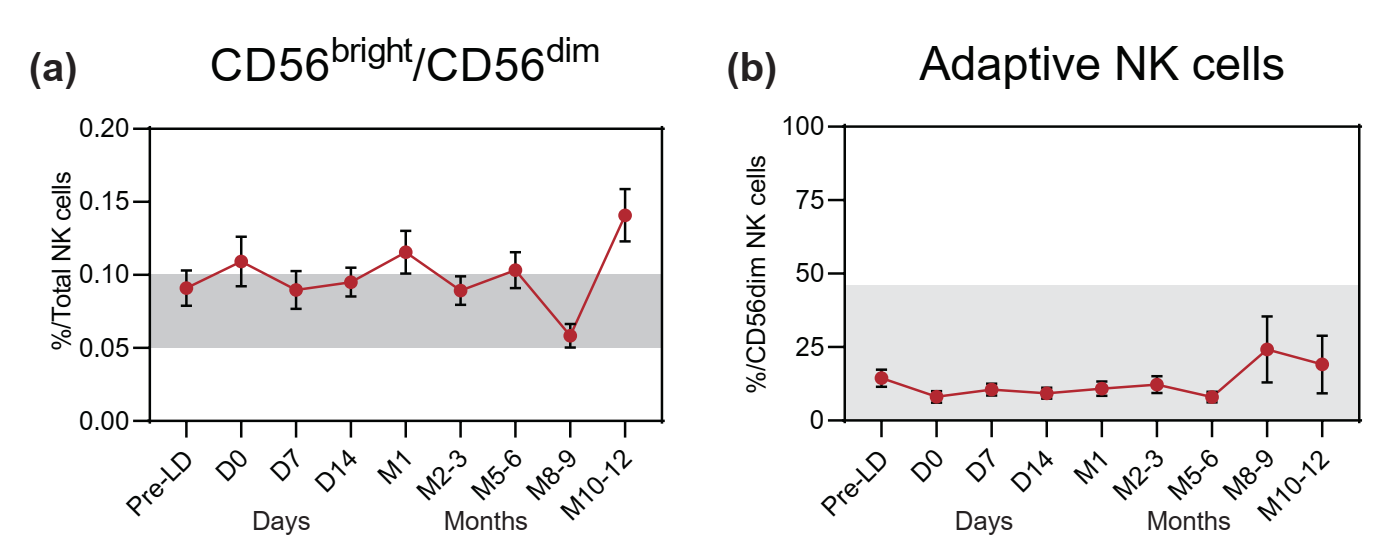


Supplemental figure 2. CD56^bright^/CD56^dim^ ratios and adaptive NK cells following CAR-T cell infusion.

**(a-b)** Longitudinal changes in the CD56^bright^/CD56^dim^ ratio among total NK cells (a) and adaptive NK cells (b), from pre-lymphodepletion (pre-LD) to one year after CAR-T cell infusion.

**Supplemental figure 3**





Supplemental figure 3. NK cell reconstitution in cancer and SLE patients.

**(a)** Numerical recovery of NK cells in cancer and SLE patients. **(b)** Phenotypic recovery of NK cells in cancer and SLE patients. Blue asterisks indicate statistically significant differences between the cancer group and the normal reference range; gray asterisks indicate differences between the SLE group and the normal reference range. **(c)** Proportion of adaptive NK cells in patients with NHL, B-ALL, and SLE. Black asterisks indicate differences of proportion of adaptive NK cells between the NHL and the SLE group. *P < 0.05; **P < 0.01; ***P < 0.001; ****P < 0.0001.

NK, natural killer; SLE, systemic lupus erythematosus; LD, lymphodepletion; D, day; M, month; NHL, non-Hodgkin lymphoma; B-ALL, B-cell acute lymphoblastic leukemia.

**Supplemental figure 4**


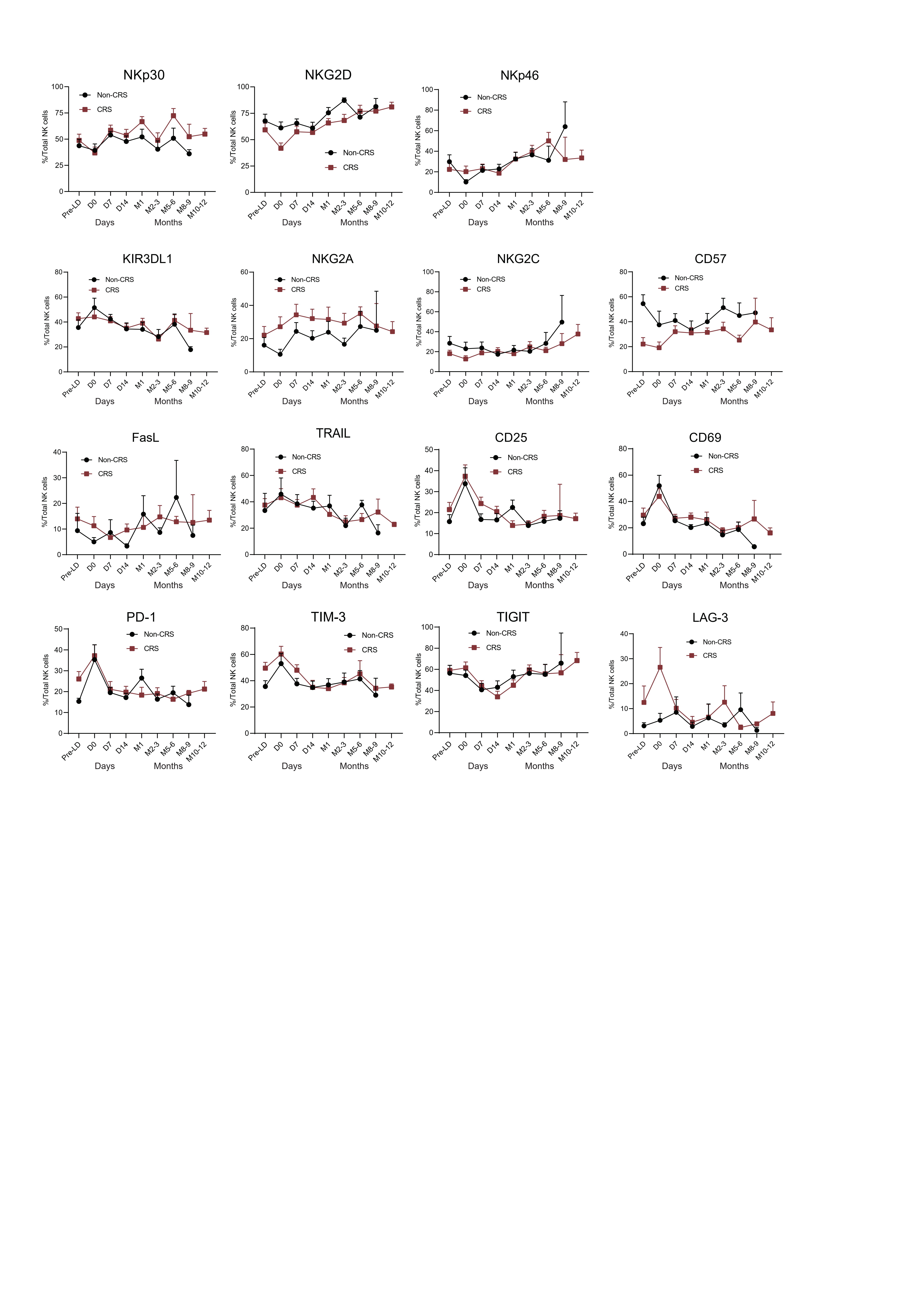


Supplemental figure 4. Dynamics of NK-cell markers between CRS and non-CRS groups.

Changes in the expression of NK-cell markers over time between patients with and without CRS.

LD, lymphodepletion; D, day; M, month; NK, natural killer; CRS, cytokine release syndrome.

**Supplemental figure 5**


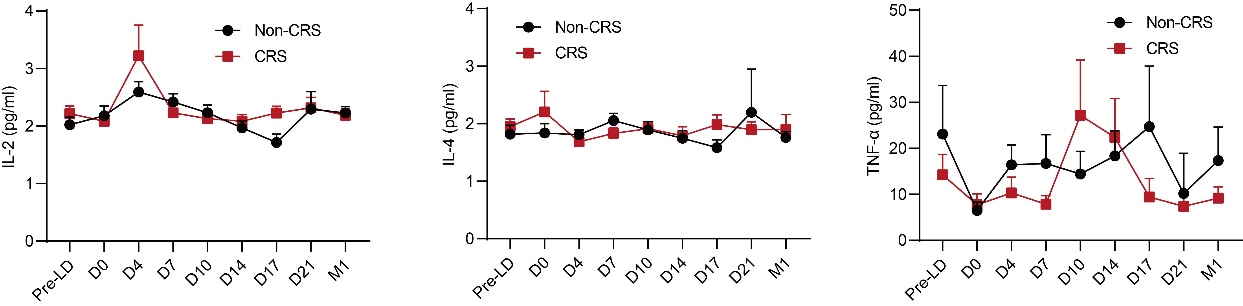


Supplemental figure 5. IL-2, IL-4, and TNF-α levels in CRS and non-CRS groups.

Plasma levels of IL-2, IL-4, and TNF-α over time in patients with and without CRS.

IL, interleukin; TNF, tumor necrosis factor; LD, lymphodepletion; D, day; M, month; CRS, cytokine release syndrome.

**Supplemental figure 6**


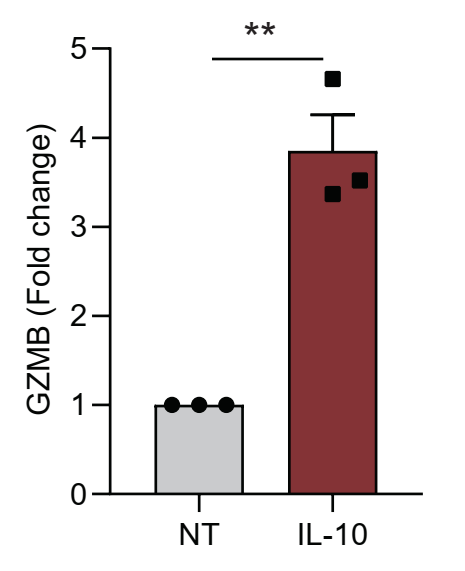


Supplemental figure 6. Granzyme B expression in NK cells with or without IL-10 treatment.

Expression of granzyme B (GZMB) in NK cells treated with IL-10 compared to NT controls detected by RT-qPCR. Statistical analysis was performed using unpaired Student’s t test. ** P<0.01.

IL, interleukin; NK, natural killer; NT, non-treated condition; GZMB, granzyme B; RT-qPCR, real-time quantitative polymerase chain reaction.

**Supplemental figure 7**


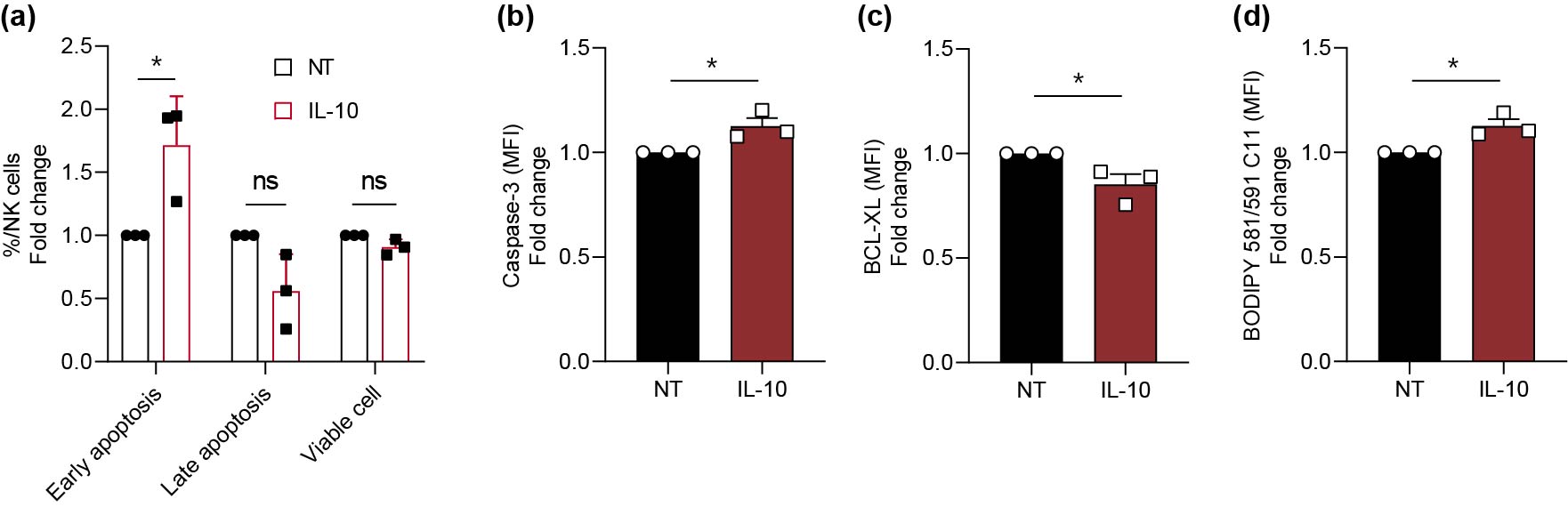


Supplemental figure 7. Apoptosis, caspase-3, BCL-XL, and lipid peroxidation levels of NK cells derived from patients treated with or without IL-10.

**(a-d)** Apoptosis (a), caspase-3 (b), BCL-XL (c), and lipid peroxidation (d) levels in NK cells treated with or without IL-10. NK cell samples were collected from three patients: one at the M1 time point from a patient who had experienced CRS, one at the M1 time point from a patient without CRS, and one from apheresis prior to CAR-T cell preparation. NK cells isolated from these samples were activated and expanded using the IL-21 NK Cell Expansion Kit, followed by IL-10 or control treatment for 48 hours. Subsequently, apoptosis, caspase-3, BCL-XL, and lipid peroxidation were assessed. Statistical analysis was performed using unpaired Student’s t test. * P<0.05; ns, not significant.

NT, non-treated condition; IL, interleukin; NK, natural killer.

**Supplemental figure 8**


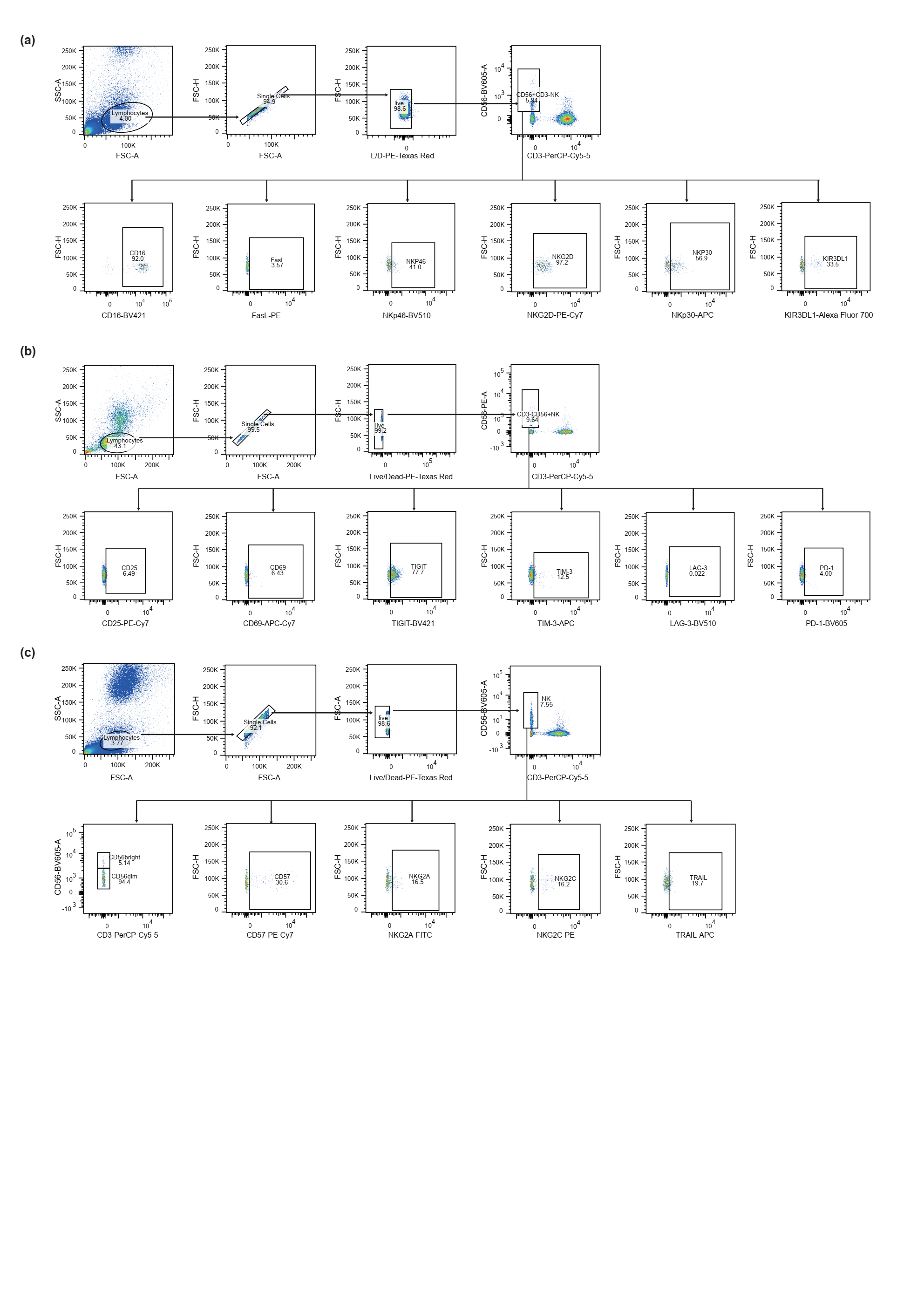


Supplemental figure 8. Gating strategy.

**(a)** Gating strategy for the panel used to detect NK cell surface markers CD16, FasL, NKp46, NKG2D, NKp30, and KIR3DL1. **(b)** Gating strategy for the panel used to detect NK cell activation and inhibitory markers CD25, CD69, TIGIT, TIM-3, LAG-3, and PD-1. **(c)** Gating strategy for the panel used to detect NK cell subsets and differentiation markers, including CD56^bright^, CD56^dim^, CD57, NKG2A, NKG2C, and TRAIL, as well as subpopulations defined by NKG2A and CD57, NKG2A and NKG2C, and CD56^dim^NKG2C⁺CD57⁺ NK cells. L/D, live/dead.

**References**

1. Wu J, Tang L, Du M, et al. Plasma proteomic and metabolomic signatures of B-ALL patients during CAR-T cell therapy. *Clin Transl Med*. 2023;13(3):e1225.

2. Zhou Y, Zhou B, Pache L, et al. Metascape provides a biologist-oriented resource for the analysis of systems-level datasets. *Nat Commun*. 2019;10(1):1523.

3. Kennedy PR, Arvindam US, Phung SK, et al. Metabolic programs drive function of therapeutic NK cells in hypoxic tumor environments. *Sci Adv*. 2024;10(44):eadn1849.
